# Familydemic Cross Country and Gender Dataset on work and family outcomes during COVID-19 pandemic

**DOI:** 10.1038/s41597-022-01880-8

**Published:** 2023-01-03

**Authors:** Anna Kurowska, Ilyar Heydari Barardehi, Sylvia Fuller, Richard J. Petts, Gayle Kaufman, Andrea Doucet, Cassandra Engeman, Anna Matysiak, Raffaele Guetto, Thordis Reimer, Tsegachew Degu Kasegn, Daniele Vignoli, Ann-Zofie Duvander, Shirely Gatenio Gabel

**Affiliations:** 1 Faculty of Political Science and International Studies; Interdisciplinary Centre for Labour Market and Family Dynamics (LabFam), University of Warsaw, Warsaw, Poland; 2 Department of Sociology, University of British Columbia, Vancouver, Canada; 3 Department of Sociology, Ball State University, Muncie, USA; 4 Department of Sociology, Davidson College, Davidson, USA; 5 Department of Sociology and Women’s and Gender Studies, Brock University, St. Catharines, Canada; 6 Swedish Institute for Social Research, Stockholm University, Stockholm, Sweden; 7 Faculty of Economic Sciences; Interdisciplinary Centre for Labour Market and Family Dynamics (LabFam), University of Warsaw, Warsaw, Poland; 8 Department of Statistics, Computer Science, Applications, University of Florence, Florence, Italy; 9 Faculty of Business, Economics and Social Sciences, University of Hamburg, Hamburg, Germany; 10 Mid Sweden University, Sundsvall, Sweden; Stockholm University, Stockholm, Sweden; 11 Graduate School of Social Service, Fordham University, New York, USA

**Keywords:** Society, Sociology

## Abstract

Here we present the Familydemic Cross Country and Gender Dataset (FCCGD), which offers cross country and gender comparative data on work and family outcomes among parents of dependent children, before and during the COVID-19 pandemic. It covers six countries from two continents representing diverse welfare regimes as well as distinct policy reactions to the pandemic outbreak. The FCCGD was created using the first wave of a web-based international survey (Familydemic) carried out between June and September 2021, on large samples of parents (aged 20–59) living with at least one child under 12 in Canada, Germany, Italy, Poland, Sweden, and the US. While individual datasets are not available due to country-level restriction policies, the presented database allows for cross-country comparison of a wide range of employment outcomes and work arrangements, the division of diverse tasks of unpaid labour (housework and childcare) in couples, experiences with childcare and school closures due to the pandemic and subjective assessments of changes to work-life balance, career prospects and the financial situation of families (234 variables).

## Background & Summary

The COVID-19 pandemic has been highly disruptive to work and family life. Many workers were laid off, unemployment rates increased, and workers faced reduced hours^1,2^. Further, gender gaps in employment increased as mothers decreased work hours more steeply than fathers^3,4^. Work was also transformed as an increasing number of organisations and workers adopted remote work as a strategy^5,6^. At the same time, working parents found themselves with increased childcare responsibilities during the workday as daycare centres, and schools closed^7^. Country differences in childcare policy responses had the potential to impact work-family reconciliation as well as educational outcomes^8^. As with employment, there have been notable gender differences in housework and childcare responsibilities. Several studies have shown an increase in women’s, and particularly mothers’, domestic labour during lockdowns^9,10^. Yet, there has also been evidence that an increasing number of couples shared housework and childcare equally compared to before the pandemic^11^.

Since the outbreak of the pandemic, numerous studies have been carried out that aimed at assessing the changes in the work and family lives of parents in different countries (e.g., Study on Parents’ Divisions of Labor during COVID-19 in the US, Covid19 Gender In(equality) Survey in the Netherlands, and the SParWell survey in Italy)^7,11,12^. However, the majority of them are either narrowed to single countries only^1–25^ or to limited subgroups of countries in one continent^26,27^. The result is a lack of cross-country datasets that would enable wider comparisons. The majority of pandemic-related datasets are also based on purely convenience samples, substantially deviating in structure from benchmark populations. Moreover, virtually only the short-term consequences of the pandemic have been explored (i.e., the first lockdown period and shortly thereafter), providing limited knowledge into how families adapt and thrive in new and challenging conditions over the longer term. The dimension of the current pandemic—given its duration and pervasiveness—cannot be usefully compared to short-term shocks. Furthermore, the available large cross-country datasets that cover the first year of the pandemic do not include comprehensive sets of questions that enable comparative overviews of diverse aspects of work and family lives of mothers and fathers that were affected during the COVID-19 pandemic, particularly in regard to distributions of detailed childcare and housework tasks and time children have spent at home due to diverse pandemic-related reasons. As a result, researchers face difficulties making cross-country comparisons of the work and family outcomes for parents during the COVID-19 pandemic^22–25,28^. The FCCGD^29^ presented here aims to address this gap as it enables researchers to make cross-country comparisons of diverse aspects of the work and family lives of parents of dependent children (having at least one child under 12) across six countries: Canada, Germany, Italy, Poland, Sweden and the US. The FCCGD^29^ data can also be useful for cross-validating results from previous pandemic surveys conducted in these countries^2,4,11–13,16,18,21,22,24,28,30^.

The dataset covers a wide range of work and family related topics, including:

1. Division of five different childcare related tasks in couples of partnered mothers and fathers of dependent children before the pandemic (February 2020; retrospective data) and for the moment of the survey (June-September 2021);
2. Division of seven different housework related tasks in couples of partnered mothers and fathers of dependent children before the pandemic (February 2020; retrospective data) and for the moment of the survey (June-September 2021);
3. Employment status of mothers and fathers of dependent children before the pandemic (February 2020; retrospective data) and for the moment of the survey (June-September 2021);
4. Reasons for not working among mothers and fathers of dependent children before the pandemic (February 2020; retrospective data) and for the moment of the survey (June-September 2021);
5. Minimum, maximum, median and mean number of hours worked by (self-)employed mothers and fathers of dependent children before the pandemic (February 2020; retrospective data) and for the moment of the survey (June-September 2021);
6. Access to and use of flextime among employed mothers and fathers for the moment of the survey (June-September 2021);
7. Access to, use and frequency of home office among employed mothers and fathers of dependent children before the pandemic (February 2020; retrospective data) and for the moment of the survey (June-September 2021);
8. Willingness to work from home among mothers and fathers for the moment of the survey (June-September 2021);
9. Subjective uncertainty about job stability among employed mothers and fathers for the moment of the survey (June-September 2021);
10. Time a preschool child/children in the family was/were out of usual childcare arrangement due to unavailability of childcare during the pandemic (excluding holidays);
11. Time a school-aged child/children in the family was/were out of school due to school closure during pandemic (excluding holidays);
12. Subjective change in work-life balance among (self)employed mothers and fathers during the COVID-19 pandemic;
13. Subjective change in career prospects among mothers and fathers during the COVID-19 pandemic;
14. Subjective change in financial situation among mothers and fathers during the COVID-19 pandemic.

It should be noted that any data that refers to an earlier period than the moment of the survey is retrospective and therefore relies on the memory of respondents about the events that happened up to even 1.5 years before the data collection. Therefore, these responses must be treated with caution.

**Fig. 1:**
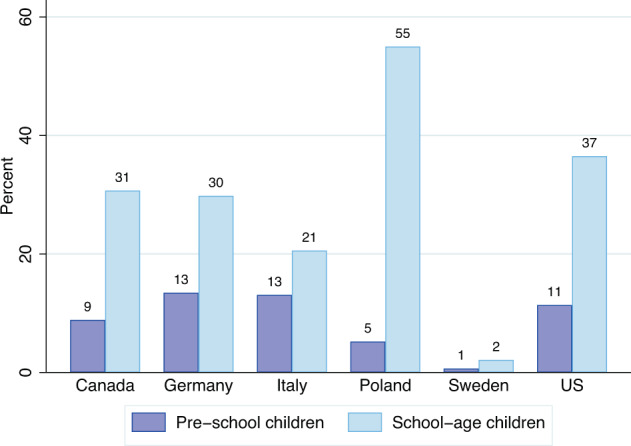
Shares (in percents) of parents with at least one child at home for six months or more as a result of childcare unavailability/school closures or classes being moved online due to the COVID-19 pandemic, by country (period from March 2020 till June 2021, excluding holidays; data source: Familydemic Cross Country and Gender Dataset).

**Fig. 2:**
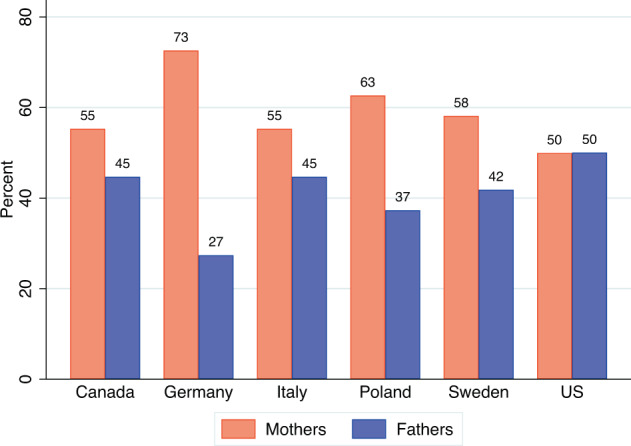
Shares (in percents) of mothers and fathers at the moment of the survey (June-September 2021) who reported spending more time on childcare than before the COVID-19 pandemic (February 2020), by country (data source: Familydemic Cross Country and Gender Dataset).

**Fig. 3:**
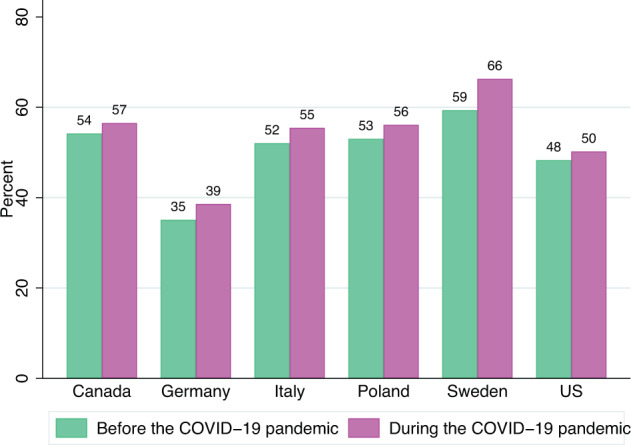
Shares (in percents) of mothers reporting a non-gendered (non-traditional) division of child care oversight before the pandemic (February 2020) and in the moment of the survey (June-September 2021), by country (data source: Familydemic Cross Country and Gender Dataset).

**Fig. 4:**
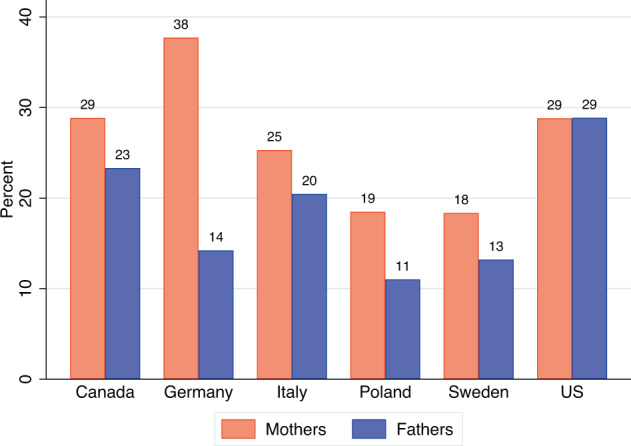
Share (in percents) of employed mothers and fathers who would like to work from home in the future, by country (data from June-September 2021; data source: Familydemic Cross Country and Gender Dataset).

**Fig. 5:**
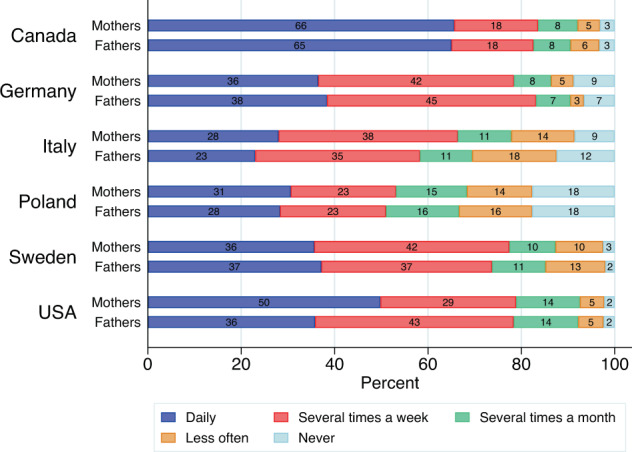
Distribution of the frequency of working from home among those mothers and fathers who at the moment of the survey (June-September 2021) had access to home-based work, by country (data source: Familydemic Cross Country and Gender Dataset).

For each question with categorical responses we provide shares of each response for the population of mothers, fathers and parents in total by country to whom the question was addressed. For each numerical question, we provide descriptive statistics—minimum and maximum values, the median, the mean, and standard deviation—for each country and by gender. For each question we also report the share of missing values. The FCCGD^29^ consists of 234 variables in total. The five figures below (Figs. 1–5) show exemplary cross-country (and gender) comparisons using just six variables from this dataset.

## Methods

### Creation of the FCCGD

The FCCGD^29^ has been created using the Familydemic Harmonised Dataset (hereafter FHD), i.e., the harmonised individual data collected via the Familydemic Survey (https://familydemic.wnpism.uw.edu.pl/about/) between June 2021 and September 2021. The survey was conducted on large quota random samples of mothers and fathers living with at least one child under 12 years old from national, certified opt-in panels in all six countries under the study. In order to adjust for deviations from the benchmark population statistics, we have used post-stratification (analytical) weights to calculate the shares of responses for each categorical variable and descriptive statistics for each numerical variable in FCCGD^29^. Weights were calculated based on Random Iterative Method (RIM weighting), using country specific structures of the population of parents with at least one child under 12, according to gender, age and education, derived from: the European Union Labour Force Survey (EU-LFS) 2020 data for Germany, Poland and Italy; from the 2020 Euromod data for Sweden; from the 2016 Census for Canada; and from the 2021 Current Population Survey (CPS) for the US.

### Familydemic Survey design and testing

The development of the Familydemic Survey was a collective endeavour, initiated and led by Anna Kurowska (University of Warsaw) and carried out between April 2020 and May 2021. The international team of researchers, who worked on the development of the final English version of the questionnaire, included Anna Kurowska, Anna Matysiak (University of Warsaw), Sylvia Fuller (University of British Columbia), Andrea Doucet (Brock University), Ann-Zofie Duvander (Stockholm University), Cassandra Engeman (Stockholm University), Richard J. Petts (Ball State University), Gayle Kaufman (Davidson College), Thordis Reimer (University of Hamburg), Shirley Gatenio Gabel (Fordham University) and in later stages also Daniele Vignoli (University of Florence) and Raffaele Guetto (University of Florence). Several other researchers helped our team develop the questionnaire between September 2020 and April 2021, and their names appear in the Acknowledgements section.

Several questions were designed on the basis of diverse existing, well-established studies from before the pandemic, covering a large array of questions related to the work and family nexus, such as: Generations and Gender Survey, International Social Survey Programme, European Social Survey, European Values Study, World Values Survey, Understanding Societies, GESIS Panel, Statistics Canada’s Longitudinal International Survey of Adults, and Canadian Quality of Work and Economic Life Study. Also, recent academic discussions on using retrospective questions in COVID-19 studies were formative in our thinking about the type of questions being used in the Familydemic Survey^31^.

The English version of the questionnaire was translated into national languages by a trained academic translator in Italy and a professional translating service in Poland; then, the translations were double-checked by the national team members. The Swedish team included bilingual and native speakers in both English and Swedish who worked together to translate the survey instrument and responses with help also from graduate research assistants. In Germany, the questionnaire was translated by Thordis Reimer and double-checked by two other researchers, as well as in an online translation tool (Deepl). In Canada, the survey was administered in both official languages; English and French. The English version of the survey was translated into French by the Angus Reid Group with assistance from Sophie Mathieu. In all countries that translated the questionnaire to a national language, the phrasing of specific questions were checked using existing translations in other international surveys that also used these questions.

We have applied two-stage testing of the final version of the questionnaire. Initially, the English version of the questionnaire was tested on a group of approximately 15 researchers from the field of work and family studies across countries in the study. Then, the translated versions of the questionnaire were tested on convenience samples of parents—up to 20 in each country. Some countries added a small number of additional country-specific questions to their national versions of the Familydemic Survey.

### Recruitment of respondents in the Familydemic Survey

Each of the participating countries hired a certified national research agency to acquire an appropriate national sample of respondents with dependent children. The agencies used quota random sampling (random samples with additional quotas applied to secure adequate representation of the population in the sample by crucial socio-demographic characteristics) from large and constantly verified (opt-in) panels. Canada, Germany, Sweden and the US each aimed at acquiring a sample of a minimum of 2,000 respondents per country, from the group of parents aged 20–59, with at least one child aged under 12 (including single parents). Poland and Italy aimed at sampling a minimum of 7,000 respondents respectively from the adult population aged 20–59. The samples from Poland and Italy consisted of parents with children under 12 (at least 2,000 in each country), parents with at least one child aged 12 or more, and the childless. However, the data referring to these two latter groups are not included in the FCCGD^29^ because comparative data does not exist for the other countries.

In Canada, the Angus Reid Group ran an online survey with 4,685 participants (parents with at least one child under the age of 12) from August 20 to September 6, 2021. Survey respondents came from Angus Reid’s opt-in online panel (the Angus Reid Forum) of Canadians, which is recruited through targeted ads and partnerships with non-governmental and charitable organisations to ensure diversity, and subject to frequent verification checks for identity, contact information, and demographic characteristics. A sample matrix was created that was designed to be representative of the population, with additional quotas to ensure minimum targets were reached for the province of residence, LGBTQ, visible minority (non-Indigenous respondents identifying as non-white), and Indigenous, Black, South Asian, and Asian respondents. Participants from this matrix were then randomly selected to receive email invitations to complete the survey, receiving a small monetary incentive for doing so. Recruitment in Canada’s three northern Territories (the Yukon, Nunavut, and Northwest Territories) was attempted via telephone recruitment, but it proved to be too difficult because of the length of the survey, recruitment challenges, and cost. Therefore, the sample is limited to ten provinces, which is typical in Canadian online surveys.

In Germany, the online survey was operated by LimeSurvey, a free online survey software. The team hired Respondi, an ISO-certified opt-in online panel provider with a large and diverse pool of people willing to take part in online surveys, who are remunerated for doing so. Respondents were selected according to information on panelists (according to the sample criteria). A sample of 2,500 was agreed, limited to parents who live in Germany and have at least one child under the age of 12 (2,445 questionnaires were returned). Quotas were set in terms of gender (50/50), age, education and German federal state residence shares in population. Programming and hosting of the main questionnaire and delivery of the screening and quota questions were carried out by the WiSo Research Laboratory at the University of Hamburg. The survey was conducted in June 2021. The data set for Germany contains 2,426 respondents.

In Italy, data were collected between July and September 2021 by the national survey company Demetra, which is well-known in Italian academic circles for its high-quality and rigorous data collection. A sample of 5,000 people aged 20–59 plus an oversampling of approximately 2,000 parents with at least one child under 12 was agreed. The online sampling scheme imposed national quotas by age group, gender, education, macro-region of residence, presence of children and age of the youngest child (N = 7,080). Quota sampling ensures that the final sample is virtually distributed as the country benchmark given by the statistics provided by the Italian National Institute of Statistics.

The Swedish team hired Novus, a major and reputable survey company in Sweden. Through Novus, they obtained an online panel, where panelists are randomly selected for surveys and proportioned according to the general population. Novus maintains a panel of over 50,000 randomly recruited panel members and is generally representative regarding age, gender and region. For the Familydemic Survey, a random sample of respondents aged 20–59 having at least one child under 12 was selected and a total of 2,683 responses were received. The survey was fielded from the 15th to 29th of September 2021, which is one month after schools and preschools had opened after the summer holidays. Respondents received a small incentive by participating in collecting points towards gift certificates.

In the US, the survey was conducted in partnership with Qualtrics. Qualtrics hosts an opt-in online panel of over 100,000 survey panelists. To ensure high-quality data, panelists must regularly update their information, are restricted in the number of surveys they can take, and are monitored in their responses to socio-demographic questions for consistent responses. Qualtrics randomly selects and proportions panelists according to the general population to provide as representative of a sample as possible. Quota constraints included gender, education, and race. The survey was fielded in July 2021, and the study was restricted to parents living in the United States with at least one child under age 12. A total of 2,383 parents completed the US survey.

In Poland, the survey was conducted in cooperation with the National Research Panel Ariadna (*Ogólnopolski Panel Badawczy Ariadna*) which hosts an opt-in online panel of over 150,000 active panelists aged 15 and over, with verified profiles. The National Research Panel holds a certificate from the Interviewer Quality Control Programme (*Program Kontroli Jakości Ankieterów*) and works according to the standards of ICC/ESOMAR International Code on Market and Social Research. A quota random sample of respondents was selected from the online panel. Quota constraints included age, gender, education and area of residence. A total of 11,183 respondents aged 20–59 completed the Polish survey in the second half of June 2021, out of which 4,188 were parents of children aged under 12.

In Table 1 below, we summarise the sample size of parents with at least one child below the age of 12 for each country, the proportion of data that was included after harmonisation in the Familydemic Harmonized Dataset (after all cleaning and harmonising procedures were performed), the means and standard deviations for age and number of children, gender ratio and the share of highly educated respondents (with Master’s degree or higher) in the sample (with no weights applied).

**Table 1:** Sample basic characteristics by country (unweighted data from the Familydemic Harmonized Dataset).

Country	Original sample size of parents	Percent of sample included in harmonised FHD	Ratio of women to men	Age Mean/SD	Number of children Mean/SD	Percent of highly educated
Canada	4,685	95.13%	1.4	38.2 (6.4)	1.93 (0.94)	18.13
Germany	2,445	85.56%	2.43	38.7 (7.1)	1.76 (0.77)	21.28
Italy	3,054	96.76%	1.03	40.8 (10.2)	0.82 (0.95)	17.18
Poland	4,188	84.12%	1.19	40.1 (11.1)	0.79 (0.95)	33.22
Sweden	2,683	98.7%	1.19	39.5 (6.3)	1.96 (0.8)	30.24
US	2,383	96.43%	1.03	35.2 (8.2)	1.96 (1.07)	19.09

Source: own calculations. Notes: original sample size of parents with at least one child under 12 - number of returned questionnaires from parents in each country before any cleaning was performed on the data; highly educated - respondents with Master’s degree or higher.

### Ethical compliance

The Familydemic Questionnaire and study protocol were approved by the Ethical Committee at the University of Warsaw (*Komisja Rektorska ds. Etyki Badań Naukowych z Udziałem Człowieka*) as well as Davidson College’s Institutional Review Board, the Swedish Ethics Review Authority (*Etikprövningsmyndigheten*), the Behavioural Review Ethics Board at the University of British Columbia, the Social Science Research Ethics Board at Brock University, the Social Ethics Board at Dalhousie University, the Research Ethics Board at the University of Guelph and the Ethical Committee (*Commissione Etica*) of the University of Florence.

The collection of the Familydemic Survey data complies with all ethical regulations of public opinion survey data. Participants have been informed of the purpose of the study and consented to share their data. Individual data has been entirely anonymized. All respondents agreed to the respective privacy policy before they answered the questionnaire.

**Fig. 6:**
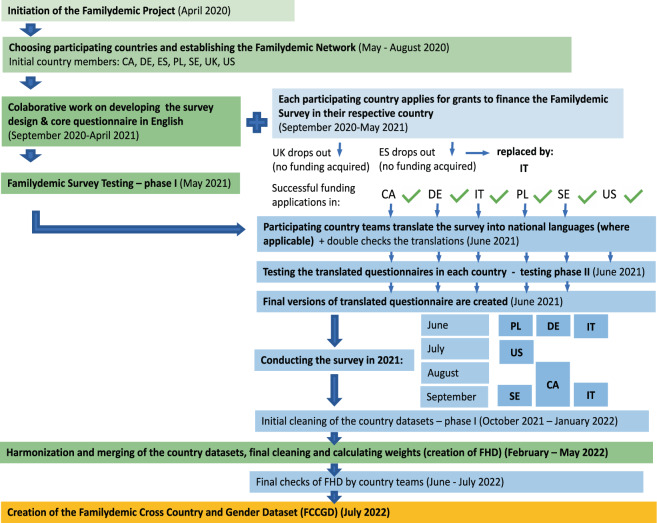
Graphical representation of the process from the establishment of the Familydemic Project to creating the Familydemic Harmonized Dataset and then the Familydemic Cross Country and Gender Dataset^29^.

The individual data from the Familydemic Survey is stored on secured servers at the universities and research agencies that participated in the project. The individual datasets, as well as FHD, due to restrictions in several countries (including Sweden, with the most restrictive policies) cannot be provided in Open Access to the public. However, the FCCGD^29^ has been allowed to be made accessible in Open Access format to researchers worldwide. Figure 6 shows the entire process from initiation of the Familydemic Project to the creation of FCCGD^29^.

## Data Records

The raw dataset presented here (FCCGD^29^) is available in open access (under CC-By Attribution 4.0 International licence) in xls format to be downloaded from the Open Science Framework (OSF) platform (direct link: 10.17605/OSF.IO/MJFZ3).

## Technical Validation

We applied a two-stage process of data cleaning in order to arrive at a reliable FCCGD^29^.

First, each country’s individual dataset was cleaned by the company who had run the survey in that particular country and also by the country team of researchers responsible for collecting data following the instructions of the coordinator of the Familydemic Project. All country datasets were checked for errors and inconsistencies. All country teams flagged the missing data using two categories: *justified missings* in cases when respondents were purposely not the addressees of the question (as a result of purposely and consistently applied filters in the online Familydemic Questionnaire) and *unjustified missings* in cases when respondents should have responded to a particular question and did not do it for different reasons.

Second, the Polish team was responsible for comparing all initially cleaned country datasets, harmonising and merging them into FHD. We identified six issues at this stage:

1. Some country samples included some respondents that were out of the target population - e.g., were older than 59 or younger than 20 or had the youngest child older than 12 (very rare cases);
2. Some differences in how variable values were coded were found (e.g. in one country Yes/No answers were coded as 1/0 and in others as 1/2);
3. Some countries were missing some of the questions from the Familydemic Questionnaire and some had included additional country-specific questions;
4. There were some differences in the sets of response options provided for respondents in some countries;
5. In the US some questions were not made obligatory for respondents and this resulted in some unjustified missings in answers;
6. In some countries filters were different than in others for some questions, resulting in unjustified missings.

To arrive at a reliable cross-country comparative dataset (FCCGD^29^) that would be comparable across countries, we decided to deal with these problems in the following ways:

1. The respondents from outside of the target population were dropped from the sample. In this regard 9 observations in Germany were dropped because respondents were older than 59 and 1 observation in Sweden was dropped because this person was younger than 20.
2. The issue of differences in the coding of variable values was easily resolved by careful recoding (harmonisation) of variables and double-checking this process with country teams.
3. The variables which were entirely missing in one of the countries and could not be created using other information from the country data, were not included in the FCCGD^29^. Some missing variables were retrieved using data from other questions. For example, we faced missing data on the pre-pandemic partnership status of respondents in some countries, but we were able to handle this missing information using information on respondents’ current marital status and the information from a separate question that asked respondents if their partnership status had changed since the onset of the pandemic.
4. In the case of a diverging set of answers for a particular variable we checked carefully if the proper set of answers could be derived for each country by merging some answers. It was possible in many cases as diverging versions of answers were usually the result of one of the countries providing respondents with more detailed responses than in other countries. One of the examples is the question about the highest achieved educational level of respondents - in Germany the set of answers contained more detailed categories. The variables for which responses could not be harmonised are not included in the FCCGD^29^.
5. We decided not to include variables with significant numbers of unjustified missings in some countries in the FCCGD^29^ as we could not handle this problem post factum.

As a result, the presented FCCGD^29^ consists only of the data retrieved from fully comparable variables in FHD across all participating countries without any non-random and significant number of missing values.

The variables included in FCCGD^29^ should not be confused with the variables included in FHD, although they are directly related through the questions they refer to from the Familydemic Survey.

In the file with FCCGD^29^ available at the OSF platform, for the sake of transparency, we also provide the sample characteristics by country according to crucial socio-demographic characteristics (before applying weights to the data): gender, age, education, labour market status and ISCO-08 1 digit code (major group) for occupation.

## Usage Notes

The data describe key work and family indicators by gender and country, providing a plethora of opportunities to explore multiple facets related to work and parenting during the pandemic. The dataset is valuable for scholars interested in accessing key information about work and family experiences and comparing these experiences across genders in the particular countries included. The availability of identical indicators collected at a similar time across a diverse range of countries that vary in their institutional arrangements and pandemic responses also facilitates unique cross-national comparisons not possible with other data sources. The data can potentially be used to study numerous issues related to work-family experiences of parents of young children during the pandemic, including but not limited to the following:

1. Mothers and fathers’ pre-pandemic perceptions of parental divisions of five childcare tasks and seven housework tasks to divisions later in the pandemic. While numerous studies documented changes in household divisions of labour in the first wave of the pandemic^30,32,33^, there has been less research documenting divisions later in the pandemic. Data from this later time-point will be valuable for scholars interested in assessing the persistence of early-pandemic shifts in particular countries and comparing across countries. The potential research questions to be addressed using FCCGD^29^ could be for example: How has the increase in time spent on childcare and on household tasks during the pandemic differed across countries (United States, Canada, Germany, Italy, Sweden and Poland) and gender for parents of children under 12? Have the changes in the division of household and childcare responsibilities in the longer perspective (over a year) been similar or different across diverse countries in Europe (Germany, Italy, Sweden and Poland) and North America (the United States & Canada)? Which specific tasks became more equally (less equally) distributed and how? What are the gender differences across these countries in the perception of changes to the division of unpaid labour in the family among parents of children under 12?
2. Comparisons of the nature and degree of changes in employment patterns, including changes in hours of work, employment status (e.g. employed, on-leave, unemployed) and working from home. While good quality data can be found on these indicators for some individual countries, operationalization of indicators varies, making cross-country comparisons problematic. Our data provide a unique source of harmonised data on these key variables collected during a common time frame. Information on employment patterns can in turn be contextualised with comparisons of differences across countries in reported barriers to employment and pandemic-related disruptions to schooling and childcare arrangements. Some potential research questions to be addressed using FCCGD^29^ include: How has the mean number of hours worked changed after about 1.5 years since the outbreak of the pandemic among parents of children under 12, across genders and countries (United States, Canada, Germany, Italy, Sweden and Poland)? Has the frequency of working from home among those with access to it already before the pandemic increased during the pandemic across countries (United States, Canada, Germany, Italy, Sweden and Poland) and genders and between employees and self-employed parents? How did the perception of job insecurity differ among employed mothers and fathers of children under 12 in the United States, Canada, Germany, Italy, Sweden and Poland in mid-2021?
3. Indicators of family and individual well-being including perceived changes in financial situation, perceptions of career prospects, and work-life balance in June-Sept 2021 compared to before the pandemic. Comparable indicators on these important aspects of well-being can add insight into the consequences of cross-country and gender differences in changes to work-family and care arrangements. The potential research questions to be addressed using FCCGD^29^ could be for example: How many working fathers and mothers of children under 12 across countries (United States, Canada, Germany, Italy, Sweden and Poland) experienced deterioration in their work-life balance during the pandemic? How does the gender gap in career prospects for working mothers and fathers of children under 12 differ across countries?
4. Incidence and frequency of children in pre-school and school-age staying at home due to lockdowns and school closures during the pandemic. This data provides comparative information on the potential childcare and schooling burden experienced by parents in the six countries due to the pandemic-related lockdowns. The potential research questions to be addressed using FCCGD^29^ could be for example: What share of parents of children under 7 in different countries (United States, Canada, Germany, Italy, Sweden and Poland) experienced a lack of access to childcare that they usually used during the first 15 months of the pandemic? How long were their children out of childcare due to childcare closures related to the pandemic? How long was at least one child at school age at home due to school closures during the pandemic in different countries (United States, Canada, Germany, Italy, Sweden and Poland)?

It is important to note that the countries included in the dataset vary in their institutional arrangements, with sub-national jurisdictions determining key responses in some countries. In Canada, for example, school closures varied across and within provinces such that country-level data on time children were out of school due to closures will not perfectly capture the situation in any particular area, but rather provides an aggregate picture of outcomes that vary across regions.

While obviously not as versatile as full microdata, tabular data derived from surveys can provide critical descriptive information to researchers, policy-makers, and other members of the public. Indeed, the value of providing open access to aggregated cross-tabular data on key variables is highlighted by the fact that major statistical agencies around the world commonly devote substantial resources to providing this type of data access (e.g. Statistics Canada’s CANSIM tables, OECD or Eurostat’s Data Browsers).

Finally, the FCCGD^29^ data can also be useful for cross-validating results from previous pandemic surveys conducted in the six countries covered by our survey.

## Data Availability

The STATA do-file with codes used to create tables needed for creation of the presented dataset (FCCGD^29^) from FHD is available at 10.17605/OSF.IO/MJFZ3. The process of harmonisation and merging of the country’s individual datasets in order to create FHD has been recorded in a STATA do-file and is available upon request from Anna Kurowska. We used STATA 17 for this purpose. Due to country-specific policy restrictions, the FHD cannot be made available to the public.
